# Perspectives of telemedicine-based services among family caregivers of patients with end-of-life cancer: a qualitative study in mainland China

**DOI:** 10.1186/s12904-024-01347-0

**Published:** 2024-01-12

**Authors:** Junchen Guo, Xianghua Xu, Chaoyi Liu, Ying Wang, Yongyi Chen

**Affiliations:** 1 Department of Palliative Care, Hunan Cancer Hospital, No.283, Tongzipo Road, Yuelu District, Changsha, 410006 Hunan China; 2 School of Nursing, University of South China, No.28, Changsheng West Road, Hengyang, 421001 Hunan China

**Keywords:** Telemedicine, End of life, Family caregiver, Qualitative study

## Abstract

**Background:**

Despite being driven by a strong sense of duty and familial obligation, providing care for patients nearing the end of life poses challenges for family caregivers. Telemedicine has rapidly gained traction as a transformative approach to healthcare delivery, offering an array of benefits that could be particularly valuable in end-of-life care. However, research on the perspectives of telemedicine-based services among family caregivers of patients with end-of-life cancer is limited. Therefore, this study aims to explore the perspectives and preferences of telemedicine-based services among family caregivers of patients with end-of-life cancer and provide a framework for developing and executing a tailored telemedicine-based end-of-life care program that addresses the unique needs of family caregivers in mainland China.

**Method:**

A descriptive phenomenological approach was used. Family caregivers were selected using purposive sampling at a tertiary cancer hospital. One-on-one semi-structured interviews were conducted with the participants from November to December 2022. Colaizz’s method was used to analyze the interviews.

**Results:**

Fourteen participants participated in interviews. Three themes and ten subthemes were identified: motivation to receive telemedicine services (relief from the burden of home care; access to professional health care services), supportive care needs for telemedicine services (support for symptom management; negative emotional adjustment; death education; daily life care guidance), and functional expectations of telemedicine service platforms (ease of use; real-time online guidance and response; personalized automatic reminder; targeted matching push of health knowledge).

**Conclusion:**

Family caregivers expressed interest in telemedicine-based services and identified various care needs before receiving telemedicine services. The findings of this study can help policymakers and healthcare providers develop more effective and culturally appropriate telemedicine-based service programs that can better support family caregivers of end-of-life cancer patients.

**Supplementary Information:**

The online version contains supplementary material available at 10.1186/s12904-024-01347-0.

## Background

Cancer remains a global health challenge of immense magnitude, affecting millions of individuals and their families worldwide. According to the International Agency for Research on Cancer, the worldwide number of deaths from cancer reached 9.96 million in 2020 [1]. As a serious disease, it not only inflicts considerable physical suffering on patients but also places an enormous emotional and psychological burden on their family caregivers [2].

As primary providers of informal care, family caregivers hold a pivotal position in enhancing the comprehensive care quality for patients nearing the end of life [3]. This significance is particularly pronounced in China, where family plays a central role in Chinese society, and caregiving for ailing family members, especially during the end-of-life phase, is deeply rooted in Chinese culture and traditions [4]. They often assume a range of caregiving tasks, including medication management, symptom monitoring, and psychological support [5]. However, despite being driven by a strong sense of duty and familial obligation, caregiving is not devoid of its challenges. One key challenge faced by family caregivers is the lack of adequate support and resources. Limited access to healthcare information, guidance, and professional support can hinder their ability to provide optimal care [2]. Additionally, as the incidence of cancer rises, the demand for cancer care services often outstrips the available resources, leading to disparities in care quality and access, particularly in rural and underserved areas [6]. A recent study has shown that due to inadequate professional support channels, family caregivers of end-of-life cancer patients in China often experience a profound emotional toll including anxiety, depression, and caregiver burnout [7]. The burden of caregiving can lead to increased stress and a deterioration in the caregiver's own health.

With the rapid development of global information and communication technology, telemedicine has rapidly gained traction as a transformative approach to healthcare delivery, offering an array of benefits that could be particularly valuable in the context of end-of-life care [8, 9]. Through telemedicine, caregivers can access professional guidance and support without the constraints of geographical distance, scheduling conflicts, or mobility issues. Furthermore, telemedicine can enhance the quality of caregiving by equipping caregivers with access to professional knowledge to manage patient care and their own well-being better [10, 11].

In 2022, the Chinese Health Commission issued a series of policies to encourage healthcare professionals to provide telemedicine-based services to end-of-life patients and their families [12]. However, limited research has been conducted to explore the perspectives and preferences of telemedicine-based services from the point view of their family caregivers. Understanding the perspectives and preferences of caregivers is essential for the successful implementation of telemedicine-based services in China. It can help provide a framework for developing and executing a tailored telemedicine-based end-of-life care program that addresses the unique needs of family caregivers. Therefore, this study aims to (1) understand the experiences and challenges faced by family caregivers in caring for patients with end-of-life cancer, and (2) explore the attitudes, needs and expectations of family caregivers towards telemedicine-based services.

## Methods

### Study design

This study involved a descriptive phenomenological approach using in-depth, semi-structured, face-to-face interviews conducted in November and December 2022. The Standards for Reporting Qualitative Research guided this study [13].

### Participant recruitment

Purposive sampling of family caregivers of patients with end-of-life cancer attending a tertiary cancer hospital in China was undertaken. The inclusion criteria for participants were: (1) undertake primary care work equal to or exceeding four hours daily; (2) being aware that the patient has terminal-stage cancer and (3) willing to sign informed consent form. Participants were excluded if they had communication or cognitive impairments.

### Data collection

Semi-structured, face-to-face interviews were conducted by JG in a private room in the hospital at the time chosen by the participants. The interviews lasted 21-36 min and were audio recorded with the consent of the participants. Before starting the formal interviews, JG conducted a brief self-introduction to build rapport with the participants and clarified the purpose, significance, and methodology of the study. The participants were informed that they had the right to withdraw from the study at any stage.

The interview script was developed based on relevant literature [14–16] and discussions with a team of experts in the field of telemedicine and end-of-life care. A pilot version was developed and subsequently pre-tested by two participants to assess the appropriateness and understanding of the questions. The script was revised slightly after pretesting (Appendix I). Table 1 presents the final scripted questions. Data collection continued until the information was saturated, and no new themes or information emerged from the interviews.

**Table 1:** Semi-structured interview questions

No	Interview questions
1	Could you please tell me some of the feelings of caring for patients?
2	What do you know about telemedicine? (If the participant is unfamiliar with telemedicine, the researchers will provide an explanation regarding its concept and functionality.)
3	If telemedicine services were available, what factors do you think would encourage you to receive telemedicine services?
4	If telemedicine services were available, what would you like to receive from your health care professionals through it?
5	If telemedicine services were available, what kind of access and form of telemedicine services would you like to have after discharge from the hospital and what kind of telemedicine service system do you want to in your mind?
6	Is there anything else you would like to add about telemedicine services?

### Data analysis

Data collection and analysis were performed simultaneously. JG and a second researcher (XX) listened to the audio records, transcribed them verbatim within 24 h, and used Colaizz’s method to analyze the transcripts [17]. First, the transcribed text was imported into the software package NVivo plus11. Statements pertaining to the research questions were extracted and coded through repeated reading. Subsequently, significant statements were assigned meanings and categorized into themes. The findings were then integrated into exhaustive descriptions of telemedicine-based supportive care needs, and the exhaustive descriptions were validated by seeking feedback from the participants. To ensure trustworthiness, the research team met regularly to discuss and refine the analysis. To increase the transparency of the interpretations, themes and subthemes are illustrated using quotations.

### Ethical considerations

This study was approved by the Ethics Committee of the University of South China (Ethics approval number: 2022–183). This study was conducted according to the principles of the Declaration of Helsinki and followed relevant guidelines and regulations. Informed consent was obtained from all participants before they participated in this study.

## Results

**Fig. 1:**
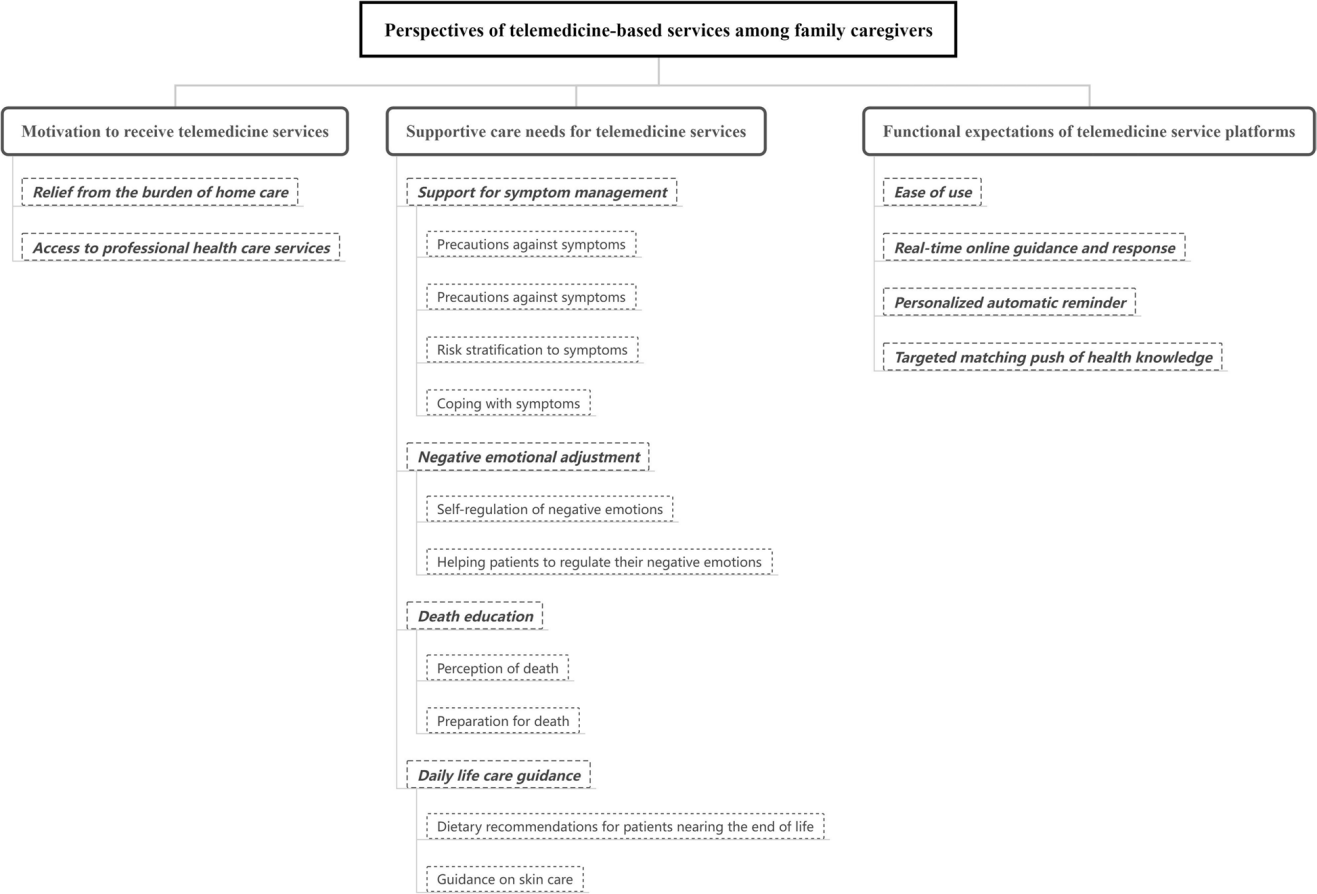
Perspectives of telemedicine-based services among family caregivers of patients with end-of-life cancer

A total of 16 caregivers were invited to participate in the study, and 14 (87.5%) completed the interviews. Table 2 lists the participants’ characteristics. Three themes and ten subthemes emerged from the thematic analysis (Fig. 1): (1) Motivation to receive telemedicine services (relief from the burden of home care; access to professional health care services), (2) Supportive care needs for telemedicine services (support for symptom management; negative emotional adjustment; death education; daily life care guidance), and (3) Functional expectations of telemedicine service platforms (ease of use; real-time online guidance and response; personalized automatic reminder; targeted matching push of health knowledge).

**Table 2:** Characteristics of participants (*n* = 14)

Variables	Categories	N (%)
Gender	Male	5
	Female	9
Age	18 ~ 39	2
	40 ~ 59	11
	> 60	1
Relationship with patient	Spouse	11
	Son or daughter	2
	Brother or sister	1
Work status	Full-time	6
	Part-time	1
	Retired	1
	Unemployed	6
Educational background	Primary school or below	1
	Junior high school	4
	Senior high school	5
	College	4
Marital status	Unmarried	2
	Married	12
Length of caring (month)	2–5	6
	7–15	3
	20–36	5

### Theme 1 Motivation to receive telemedicine services

#### Subtheme: Relief from the burden of home care

All participants expressed a high level of receptiveness towards telemedicine services. They believed that providing telemedicine services would greatly relieve the burden of caring for patients at home, allowing them to promptly seek assistance from healthcare professionals in unexpected situations they cannot handle independently.

"I think it's really useful that you have this (telemedicine), sometimes at home I can’t handle some unexpected situation. With the telemedicine, I can easily ask you online, then my stress will be relieved …" (Participant 10).

"It would be very helpful if you could provide telemedicine services so we could connect online, and I would be able to ask you what exactly to do if he (patient) faces some situation at home, and my psychological stress would be relieved…" (Participant 12).

#### Subtheme:Access to professional health care services

The participants stated that going back and forth to the hospital was inconvenient due to distance and pandemic, but believed that telemedicine services could overcome these obstacles by providing access to professional healthcare services regardless of time and location constraints.

"I don’t have to keep going back and forth to the hospital, especially with the current pandemic (COVID-19) situation making it even more troublesome. It is very convenient if I can get some professional care guidance after we communicate on the smartphone." (Participant 11).

### Theme 2 Supportive care needs for telemedicine services

#### Subtheme: Support for symptom management

The majority of participants reported that the patients experienced a diverse range of physical symptoms while being cared for at home and emphasized for symptom management support if telemedicine services can be implemented.

### Precautions against symptoms

Some participants expressed a desire for healthcare providers to use telemedicine to inform them in advance about precautions against symptoms based on their patients' conditions.

"I am very worried that he (patient) has an enterostomy, although it looks normal now, I am afraid that something unexpected might happen after going home…(sighs)" (Participant 12).

### Identification of symptoms

The participants stated that they had difficulty accurately identifying symptoms, such as dyspnea, in cancer patients near the end of their life. They felt helpless and panicked at home.

"I really do not know how to handle some situations, such as when he (patient) experienced shortness of breath at home. I really panicked and did not know what to do, how did the symptoms come about?" (Participant 5).

### Risk stratification to symptoms

Drug dosages like pain medications are typically tailored to the stage of the cancer patient’s disease, and the incidence and severity of adverse drug reactions often vary depending on the administered dose. Some participants suggested that healthcare providers should stratify patients’ symptoms according to their level of risk.

"She hadn't had a proper bowel movement for over 10 days while she was at home. It was only when I took her to the hospital that I discovered her constipation was likely caused by the heavy doses of painkillers she was taking. If I had known the reason earlier… (sighs)" (Participant 13).

### Coping with symptoms

Participants highlighted coping with symptoms as a primary concern. They shared that they could only use simple coping methods when their patients experienced symptoms at home. However, this approach does not alleviate the patient's symptoms.

"When he (patient) has a fever, in fact, I know that the simplest way to deal with it, but I do not seem to change his temperature, I am very worried, do not know what else I can do…" (Participant 5).

"If telemedicine is possible, I would like to get your guidance about how to alleviate his (patient) symptoms, such as pain, even if his disease is untreatable." (Participant 8).

#### Subtheme: Negative emotional adjustment

Most participants indicated that they experienced various negative emotions while providing long-term care to patients. They were eager to find channels to relieve their negative emotions.

### Self-regulation of negative emotions

Confronting the reality of incurable diseases, witnessing patients’ pain and suffering, and being unable to control their current condition cause caregivers to experience negative emotions. They urgently need a method to self-regulate their negative emotions.

"I couldn't help but go through a rollercoaster of emotions and reach my breaking point while taking care of him (patient) at home. When this critical point collapsed, I also needed to find a way to break through, so I wanted to soothe my bad emotions through telemedicine." (Participant 6).

"I feel anxious when I think of coming to the hospital. I think the physician should focus on the emotional needs of families through this telemedicine." (Participant 14).

### Helping patients to regulate their negative emotions

Two participants stated that if the patient was in a bad mood, it would also affect their mood, and they did not know how to help the patient regulate.

"The most significant pressure actually is him (patient). He knows his situation, but he does not say, I do not know how to help him relieve this emotion…. (sighs)"(Participant 8).

"Sometimes he was definitely cranky, so over time, I basically could not communicate with him, and I did not know how to calm him down, this led to my heart being very afflicted…" (Participant 2).

#### Subtheme: Death education

Death is inevitable for patients with end-of-life cancer. A small number of participants voiced their desire for support in death education, specifically regarding the perception of the death of patients and preparation for death.

### Perception of death

How to inform patients that death is inevitable was one of the main issues for some participants. They hoped that healthcare professionals could make patients correctly understand the objective natural phenomenon of death and reduce fear of death.

"Everyone's perception of death varies. When he (patient) experiences pain, he may express a wish to let go. However, the problem is that he will want all kinds of ways to survive when he feels comfortable. It makes me wonder if healthcare professionals can communicate this reality (death) to the patient using telemedicine or other approaches." (Participant 6).

### Preparation for death

During the interviews, the participants expressed a sense of helplessness when discussing death preparation topics, such as funerals and last wishes, with patients. They suggested that such conversations would be more effective if initiated by healthcare professionals.

"Whenever I try to discuss the topic of death with him (patient), he chooses to avoid it. So I think if there is telemedicine, the doctor can communicate with him about his last wishes online and other preparations for death so that I can make final arrangements for his life…" (Participant 6).

#### Subtheme: Daily life care guidance

One of the critical needs frequently expressed by participants was to provide daily life care guidance through telemedicine. Participants wanted to take care of their loved ones as much as possible.

### Dietary recommendations for patients nearing the end of life

Some caregivers expressed uncertainty about how to properly prepare meals for their patients, which left them feeling helpless.

"He (patient) wants chilli at every meal, but actually he cannot eat that. I have prepared those fruits, but he refuses to eat them as he believes those nutritious things will cause acid reflux…(sighs)" (Participant 8).

### Guidance on skin care

The participants highlighted that sometimes the patient had a foul odour and could only perform simple skin cleaning at home, but the odour did not dissipate.

"I particularly want you to help me how to clean his skin at home through telemedicine. Sometimes he smells bad, and I just simply deal with it…" (Participant 9).

### Theme 3 Functional expectations on telemedicine service system

#### Subtheme: Ease of use

Ease of use is a critical functionality that telemedicine service systems must provide. Specifically, this entails the provision of accessible tools such as user-friendly apps and WeChat interfaces with a straightforward login process.

"I would appreciate it if you provided me with an app. I would like to log into your app effortlessly using my smartphone without going through any complex procedures." (Participant 14).

"I prefer that your app be accessed directly with a simple click on WeChat, with a few steps involved…" (Participant 9).

#### Subtheme: Real-time online guidance and response

Almost all participants said that the telemedicine service system should provide real-time online guidance and responses.

"I request that your team is available for real-time online assistance in any unexpected situations. Especially if the patient has a sudden emergency at home…" (Participant 4).

"It would be greatly appreciated if this system could respond to my inquiries on time because many times I do not know what to do at home, and I need your help." (Participant 14).

#### Subtheme: Personalized automatic reminder

Participants highlighted that a telemedicine service system should provide automatic reminders based on each patient's unique condition, such as which physiological indicators have reached the warning status, so that they can remain informed about any changes or developments in the patient.

"Sometimes I have no idea when his indicators have dropped. I could have known his physical condition in real time if the system had automatic reminders." (Participant 5).

#### Subtheme: Targeted matching push of health knowledge

Nearly half the participants said they had no channel to gain health knowledge at home and were eager for a telemedicine service system to impart knowledge to them in a targeted matching manner.

"Sometimes my mother experiences sudden bouts of vomiting at home, so I hope this system can give me some basic knowledge, such as whether the vomiting is sudden or regular…" (Participant 4).

"Maybe the doctor doesn't have time, and then it is also challenging to explain the professional knowledge clearly to me. I hope that the system can be targeted to push me some knowledge and let me understand…" (Participant 14).

## Discussion

Identifying and exploring family caregivers' perspectives and preferences are essential for developing telemedicine-based services [18]. This qualitative study provides novel data, combining the perspectives of Chinese family caregivers of patients with end-of-life cancer to highlight the advantages of telemedicine services. The results of our study showed that all family caregivers embraced the opportunities that telemedicine services presented. They considered a potential telemedicine tool acceptable for relieving the burden of home care and accessing professional healthcare services. Previous studies have posited that one cause of the care burden for family caregivers is their inability to respond to emergencies when a loved one is discharged from hospital [14]. In this study, participants expressed that a distinct advantage of telemedicine was the ability to promptly access assistance from healthcare professionals in the event of unforeseen circumstances outside the hospital setting. Overcoming the limitations of time and geographic distance is another notable benefit of telemedicine, particularly for individuals residing in rural or remote areas [19, 20]. Pasanen et al. identified that telemedicine services have the potential to expand access to scarce care resources, which is similar to our results [21]. Healthcare providers should recognize the potential benefits of telemedicine in improving accessibility to end-of-life care to reduce caregivers’ stress as much as possible.

This study found that symptom management was the primary concern of family caregivers, which aligned with the findings of a previous study [22]. According to the National Institute on Aging, most end-of-life cancer patients frequently experience negative symptoms, such as high incidence of pain [23, 24]. However, owing to the limited access to professional end-of-life symptom care knowledge, caregivers face significant challenges in providing adequate symptom management after hospital discharge [25]. Telemedicine has emerged as a promising avenue to address this challenge. Healthcare professionals can provide caregivers with the necessary knowledge and skills to prevent, recognize, and effectively respond to unexpected or worsening symptoms at home through telemedicine, thereby improving their ability to manage complex end-of-life symptoms.

In this study, some participants experienced severe negative emotions while caring for the patients. They seldom expressed their feelings to their patients or other family members to avoid increasing the burden on others. Additionally, in the terminal stage of cancer, patients often experience a significant decline in physiological function and intense psychological trauma due to the fear of the unknown realm of death, and this negative psychological emotion can also subconsciously affect their caregivers [26]. Long-term accumulation of negative emotions can erode caregivers’ confidence in care and diminish their quality of life [25]. However, psychological distress among family caregivers in China is rarely assessed, and they lack access to professional avenues for emotional support. A recent systematic review showed that web-based interventions can significantly alleviate anxiety in end-of-life cancer patients [27]. In the future, healthcare professionals can build programs such as telemedicine-based psychological crisis interventions to promptly regulate the negative emotions of home caregivers and patients, thereby facilitating their readjustment to life.

Discussions regarding death or death-related issues is often negative or sensitive in traditional Chinese culture [28]. Death education can help people develop knowledge, attitudes, and skills related to death and bereavement [29]. Presently, the effective implementation of death education for patients poses a substantial challenge for family caregivers, encompassing vital subjects, such as death perception and open conversations about funeral arrangements. To address this, healthcare professionals can explore the potential of virtual reality-based games to simulate end-of-life experiences. By leveraging innovative approaches, caregivers can alleviate existing difficulties and assist patients in confronting the concept of death more comfortably [30]. The need for daily life care guidance support was mentioned several times in our study, specifically in areas such as diet, and skin care. Most participants preferred to receive professional daily life care guidance from healthcare providers through telemedicine, as it could help them avoid mistakes in caring for patients and improve their quality of life. Sin et al. [31]. found that family caregivers prefer flexible, self-directed, web-based daily life care knowledge resources over traditional outpatient health education methods, which inspired the implementation of telemedicine services to take this into account and provide personal care guidance for caregivers.

The motivation to complete and continue the use of telemedicine-based services is crucial to obtain an effect [32]. Most participants in this study expressed interest in telemedicine-based services. However, high acceptance does not imply high uptake or adherence [33]. Owing to a natural decline in learning ability, most caregivers preferred to access telemedicine services through easy-to-use software, which is consistent with previous research highlighting the significance of intuitive design in promoting the engagement and acceptance of telemedicine technologies [16]. Furthermore, the participants consistently emphasized the value of real-time online guidance and responses in their telemedicine service expectations. Therefore, real-time communication features, such as video consultations, can be developed for designing telemedicine service systems to ensure that caregivers receive timely support and foster trust in telemedicine services. Personalized automatic reminders and targeted health knowledge emerged as crucial aspects of the participants' desired telemedicine services. By setting personalized automatic reminders, caregivers can better manage patients at home, and the provision of tailored health knowledge can empower caregivers to make informed decisions. Healthcare professionals should consider these functional expectations when designing telemedicine systems and developing personalized telemedicine service implementation paths, to increase their engagement with the system.

This study had several limitations. First, we performed a single-center study at a Chinese cancer hospital. However, perspectives of telemedicine-based services may differ for family caregivers from general or other hospitals as well as in other countries. Second, the majority of the family caregivers interviewed were women, and the results may not fully cover the male perspective on how telemedicine services could support them. Third, our study was restricted to family caregivers of end-of-life cancer patients, and the results may not be generalizable across all treatment phases. Finally, the interview questions mainly focus on the positive aspects of telemedicine and lack of discussion on the negative aspects of this model. Therefore, future studies can delve deeper into this area to comprehensively explore any perceived drawbacks, challenges, or concerns associated with telemedicine. This would provide valuable insights, allowing for a more balanced understanding of both the advantages and disadvantages of telemedicine in end-of-life care.

## Conclusion

This study offers invaluable insights into the perspectives surrounding telemedicine-based services among family caregivers of end-of-life cancer patients. Family caregivers expressed interest in telemedicine-based services and identified various care needs before receiving telemedicine services. As telemedicine continues to evolve, policymakers and healthcare providers should consider these insights to develop more effective and culturally appropriate telemedicine-based service programs that can better support family caregivers of end-of-life cancer patients in mainland China.

### Supplementary Information

**Additional file 1.**

## Data Availability

The data used during this study are available from the corresponding authors on reasonable request.

## Abbreviation

COVID-19: Coronavirus disease of 2019
