# Supplementary material for: Perspectives of telemedicine-based services among family caregivers of patients with end-of-life cancer: a qualitative study in mainland China
Source: BMC Palliat Care. 2024 Jan 12;23:16. doi: 10.1186/s12904-024-01347-0 (PMC10785499; doi:10.1186/s12904-024-01347-0)
Supplement: Supplementary file 1 — Additional file 1. [file 12904_2024_1347_MOESM1_ESM.docx]

Interview Guide-English Version

| Question 1 | Could you please tell me some of the feelings of caring for patients? |
| --- | --- |
| Question 2 | **What do you know about telemedicine?**   - Could you provide an overview of the main components or technologies commonly used in telemedicine? (If the participant is familiar with telemedicine, the researchers will use this question.) - If the participant is unfamiliar with telemedicine, the researchers will provide an explanation regarding the concept and functionality of telemedicine. |
| Question 3 | **If telemedicine services were available, what factors do you think would encourage you to receive telemedicine services?**   - Are there specific convenience factors or benefits that you believe would make telemedicine an attractive option for you? For example, you can get the information you want in a timely manner through telemedicine. |
| Question 4 | **If telemedicine services were available, what would you like to receive from your health care professionals through it?**   - Could you specify the types of healthcare services or support you would prefer to receive from your healthcare professionals via telemedicine? For example, services like symptom management or dietary guidance,etc. |
| Question 5 | **If telemedicine services were available, what kind of access and form of telemedicine services would you like to have after discharge from the hospital and what kind of telemedicine service system do you want to in your mind?**   - In what format or medium would you prefer to receive post-discharge telemedicine services, such as WeChat, phone calls or video consultations? - In your ideal telemedicine service system, what features or components do you envision as essential for meeting your healthcare needs and expectations? For example, real-time video consultations or secure messaging with healthcare providers. |
| Question 6 | **Is there anything else you would like to add about telemedicine services?**   - Are there any specific concerns or reservations you have regarding the use of telemedicine services? |
